# Persistently High Levels of Coagulation Factor XI as a Risk Factor for Venous Thrombosis

**DOI:** 10.3390/jcm12154890

**Published:** 2023-07-25

**Authors:** Luca Spiezia, Chiara Forestan, Elena Campello, Chiara Simion, Paolo Simioni

**Affiliations:** General Medicine and Thrombotic and Haemorrhagic Diseases Unit, Department of Medicine, University of Padova, 35138 Padova, Italy; chiara.forestan.cf@gmail.com (C.F.); elena.campello@unipd.it (E.C.); solidea.cs@gmail.com (C.S.); paolo.simioni@unipd.it (P.S.)

**Keywords:** hypercoagulopathy, risk factors, coagulation factor XI, thrombophilia, venous thrombosis

## Abstract

Coagulation factor XI (FXI) promotes fibrin formation and inhibits fibrinolysis. Elevated plasma FXI levels, limited to a single measurement, are associated with a higher thrombotic risk. Our case–control study aimed to identify the effect of persistently increased plasma FXI levels on the risk of deep vein thrombosis (DVT). All patients evaluated between January 2016 and January 2018 for a first episode of proximal DVT of the lower extremity were considered for enrolment. Plasma FXI levels were measured at least 1 month after the discontinuation of anticoagulant treatment (T1). The patients with increased plasma FXI levels (>90th percentile of controls) were tested again 3 months later (T2). Among the 200 enrolled patients (M/F 114/86, age range 26–87 years), 47 patients had increased plasma FXI levels at T1 and16 patients had persistently increased plasma FXI levels at T2. The adjusted odds ratio for DVT was 2.4 (95% CI, 1.3 to 5.5, *p* < 0.001) for patients with increased FXI levels at T1 and 5.2 (95% CI, 2.3 to 13.2, *p* < 0.001) for patients with persistently high FXI levels at T2. Elevated FXI levels constitute a risk factor for deep vein thrombosis, and this risk nearly doubled in patients with persistently increased plasma FXI levels. Larger prospective studies are needed to confirm our findings.

## 1. Introduction

Factor XI (FXI) is a serine protease produced by the liver that circulates in the bloodstream in its inactive form at a concentration of ~30 nMF [1]. FXI is unique among the coagulation proteases as it is a dimer of identical 80 kDa subunits that exhibit significant homology with prekallikrein [2]. FXI is cleaved into its active form FXIa by factor XIIa (FXIIa) in the presence of high-molecular-weight kininogen on negatively charged surfaces. Independent of FXIIa, FXI can also be activated by thrombin and FXIa itself. FXI is one of the components of the early phase of the intrinsic pathway or “contact phase” of the coagulation cascade [3] and contributes to the haemostatic processes mainly through thrombin generation [4]. The activation of FXI by thrombin constitutes an amplification pathway to generate additional thrombin. Thrombin has two important procoagulant functions: (a) to promote stable clot formation by converting fibrinogen into fibrin; and (b) to inhibit fibrinolysis via the activation of thrombin-activatable fibrinolysis inhibitor (TAFI), a carboxypeptidase capable of removing C-terminal lysine binding sites for tissue plasminogen activator (tPA) and plasminogen from fibrin. Interestingly, the procoagulant role of FXI may not be restricted to the activation of thrombin. Puy C et al. [5] demonstrated that FXIa may enhance FXa and thrombin generation through the inactivation of tissue factor pathway inhibitor from platelets and on endothelial cells.

Factor XI deficiency—also known as haemophilia C—is an autosomal recessive disorder that is most commonly found in the Ashkenazi Jewish population and it is generally associated with variability in bleeding phenotypes. In particular, plasma FXI levels are unable to predict postoperative or post-traumatic bleeding and most patients present mild bleeding regardless of the severity of the deficiency [6]. This perplexing observation indicates that FXI may play a supportive role in haemostasis. Interestingly, patients with congenital FXI deficiency carry a lower risk for venous thromboembolism (VTE) and ischaemic stroke, whereas high plasma FXI levels have been reported to be associated with an increased risk of both venous and arterial thrombotic complications. Using data from the Leiden Thrombophilia Study, Meijers JCM et al. [7] demonstrated an association between elevated FXI levels and risk of venous thrombotic disease. The subjects with FXI levels above 110 U/dL (upper quartile of the distribution in controls) had a twofold increased risk of venous thrombosis compared with subjects with FXI levels in the lowest quartile (<83.3 U/dL). These findings were corroborated in subsequent studies [8,9]. A study by Libourel EJ et al. [10] found that elevated plasma FXI levels may contribute to the thrombotic risk in FV Leiden carriers. A recent study reported an association between increased FXIa activity levels in patients with an acute thrombotic event and recurrent VTE [11]. Moreover, an association was also identified between elevated FXI levels and myocardial infarction, ischaemic stroke, and recurrent stroke. In particular, Merlo C et al. [12], Doggen CJ et al. [13] in a male cohort, and Berliner JI et al. [14] in a female cohort, found that increased FXI levels correlated with a twofold increased risk of myocardial infarction. A study by Suri MF et al. [15] found a threefold increased risk of ischaemic stroke, and Yang DT et al. [16] reported a fivefold increased risk of stroke or transient ischaemic attack (TIA) in patients with elevated FXI levels. Ząbczyk MT et al. [17] recently found that circulating FXIa is associated with increased risk of ischaemic stroke and cardiovascular death in patients with atrial fibrillation (AF) undergoing anticoagulant therapy during long-term follow-up. Finally, elevated FXI levels have been associated with higher risk of all-cause mortality after the first ischaemic stroke [16]. However, it bears noting that plasma FXI levels vary wildly over time and previous studies only performed a single measurement of FXI. Therefore, we endeavoured to evaluate the effect of persistently—in two consecutive determinations made at least three months apart—increased plasma FXI levels over time on the risk of DVT in the lower extremities.

## 2. Materials and Methods

### 2.1. Patients and Controls

The patients considered in the present study were part of a larger cohort that has been partially described previously [18]. All patients referred to the Thrombotic and Hemorrhagic Diseases Unit of Padova University Hospital (Italy) between January 2016 and January 2018 for a first objectively proven episode of proximal DVT of the lower extremity were considered for enrolment. DVT was confirmed by (compression) ultrasonography in accordance with the current guidelines [19]. The diagnosis of proximal DVT was established by the presence of thrombi in the iliac, femoral, and/or popliteal veins. The choice and duration of anticoagulant treatment for each enrolled patient were based on international guidelines and the treating physicians’ assessment of the individual’s risk–benefit ratio.

Exclusion criteria were (i) anticoagulant treatment < 3 months; (ii) failure to obtain informed consent; (iii) age < 18 years; (iv) antiphospholipid antibody and/or lupus anticoagulant positivity; (v) lacking thrombophilia data; (vi) pregnancy, ongoing hormonal or statin therapy, or acute medical conditions (e.g., infectious diseases, active cancer) at the time of blood sample collection. A group of healthy volunteers age- (±3 yrs) and sex-matched with cases acted as controls [20]. The following data were collected for each enrolled patient: age, sex, and body mass index—recorded at the time of the first blood sample collection—and risk factors for DVT. DVT was classified as provoked if it occurred within 3 months of exposure to exogenous risk factors including recent trauma or surgery (<3 months), immobilisation (at least 5 days), acute medical diseases, or cancer. Otherwise, DVT was classified as unprovoked. In controls, we recorded age, sex, and body mass index at the time of the first blood sample collection.

The study protocol was approved by the Ethics Committee of Padova University Hospital (4303/AO/17) in compliance with the principles of the Declaration of Helsinki.

### 2.2. Laboratory Studies

**Figure 1:**
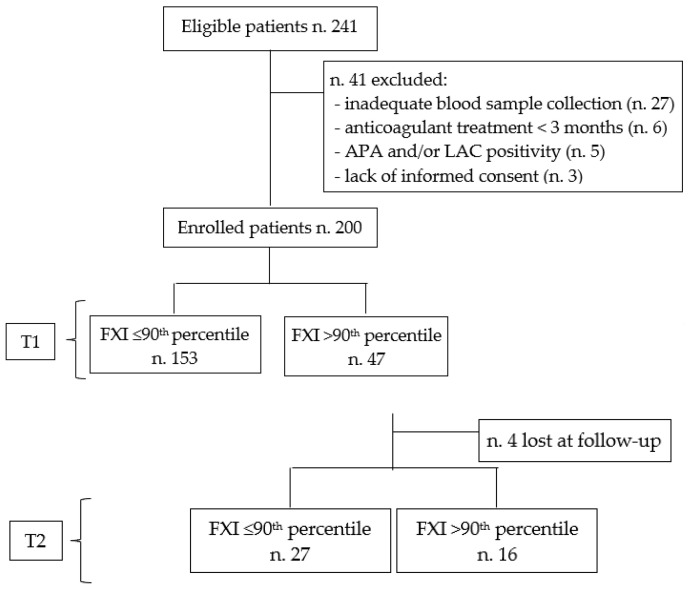
Patient flow diagram.

After obtaining informed consent, 9 mL of blood was drawn from an antecubital vein into a syringe pre-filled with 1 mL of Na-citrate 109 mM (3.2%). Platelet poor plasma (PPP) was prepared within 1 h of blood collection by double centrifugation at 1500× *g* for 10 min at room temperature. Aliquots (1 mL) were immediately frozen and stored at −80 °C until analysis. In each patient we measured FVIII and FXI activity; plasma levels of fibrinogen, D-dimer, antithrombin (AT), protein C (PC), and protein S (PS); and presence of FV Leiden mutation and prothrombin G20210A variant. In all enrolled subjects, blood samples were collected at least one month after the discontinuation of anticoagulant treatment (T1). Only patients with increased plasma FXI levels (>90th percentile of the healthy cohort) were retested at least 3 months after the previous measurement (Figure 1). AT, PC, and PS activity levels were measured as previously reported [21,22]. The criteria and cut-offs used for the classification of AT, PC, and PS defects were in line with previous studies [23,24]. Antithrombin (AT, n.v. 80–120%) activity was detected using a thrombin-based chromogenic substrate assay (Roche Diagnostics GmbH, Mannheim, Germany); protein C (PC, n.v. 80–120%) activity was measured using a commercial kit (Protein C Reagent, Siemens Healthcare Diagnostics, Milan, Italy). All tests were performed using a BCS XP coagulation analyser (Siemens Healthcare Diagnostics, Milan, Italy) according to the manufacturer’s instructions. Protein S (PS, n.v. 70–130%) activity was assessed using the ProS kit (Instrumentation Laboratory, Milan, Italy) on an ACL TOP 300 CTS coagulation analyser (Instrumentation Laboratory, Milan, Italy). Factor VIII (n.v. 60–160%) and FXI (n.v. 80–120%) activity levels were measured using specific factor-deficient plasma (Siemens Healthcare Diagnostics, Milan, Italy). Fibrinogen concentration (n.v. 150–450 mg/dL) was measured by the Clauss method using a BCT-Analyzer (Dade Behring, Marburg, Germany) according to the manufacturer’s recommendations. The D-dimer test (n.v. <500 ng/mL) was performed using a BCS XP coagulation analyser (Siemens Healthcare Diagnostics, Milan, Italy) according to the manufacturer’s instructions. Increased FVIII and fibrinogen plasma levels were confirmed in two separate samples for all enrolled subjects. Reduced anticoagulant factors levels were confirmed in two consecutive determinations and in at least 1 first-degree relative. Finally, genetic polymorphisms were determined using ABI Prism 3100 Genetic Analyzer (Applied Biosystem, Waltham, MA, USA) according to the manufacturer’s instructions.

### 2.3. Statistical Analysis

Continuous variables were expressed as a mean (±standard deviation, SD) and/or range. Categorical variables were expressed as absolute numbers and/or percentages. Mann–Whitney U and chi squared tests were used to compare differences between cases and controls. Pearson’s correlation coefficient was used to measure the statistical association between two continuous variables. Univariable and multivariable regression models were used to estimate the odds ratio (OR) with a 95% confidence interval (CI) to evaluate the contribution of elevated FXI levels to DVT risk. The odds ratios were adjusted for BMI (analysed as a continuous variable) and other thrombophilias (noncarrier vs. carrier). In all analyses, the group with plasma FXI levels below the 90th percentile of the distribution among healthy controls served as the reference for the OR. Variables included in the multivariate model were age, sex, body mass index, FVIII (>250%), fibrinogen (>600 mg/dL), protein C (<60%), protein S (<60%), antithrombin (<70%), FV Leiden (noncarrier or carrier), and the prothrombin G20210A mutation (noncarrier or carrier). All statistical analyses were performed using PASW Statistics 17.0.2 (SPSS Inc., Chicago, IL, USA) for Windows.

## 3. Results

Among the 241 eligible patients, 41 were excluded due to inadequate blood sample collection (n 27); anticoagulant treatment < 3 months (n 6); antiphospholipid antibody and/or lupus anticoagulants positivity (n 5); and lacking informed consent (n 3). Overall, 200 patients (M/F 114/86, age range 26–87 years) were included in the study. The most frequent localisation of DVT was the femoral and/or iliac veins (n 112, 56%), and 90 (45%) patients had an unprovoked event. The main characteristics of the study population are reported in Table 1. The prevalence of obesity was significantly higher in cases (n 56, 28%) than in controls (n 34, 17%; *p* < 0.01). The prevalence of thrombophilia was significantly higher in cases (n 58, 29%) than in controls (n 33, 17%; *p* < 0.01). The most common thrombophilia observed in cases was FV Leiden (n 14, 24%), followed by prothrombin G20210A mutation (n 11, 19%). Ninety-two cases (46%) experienced unprovoked DVT. The most common risk factor for provoked DVT was trauma or surgery (n 28, 31%), followed by immobilisation (n 26, 28%), cancer (n 22, 24%), and acute medical diseases (n 16, 17%).

### 3.1. T1—At Least 1 Month after Discontinuation of Anticoagulant Treatment

**Figure 2:**
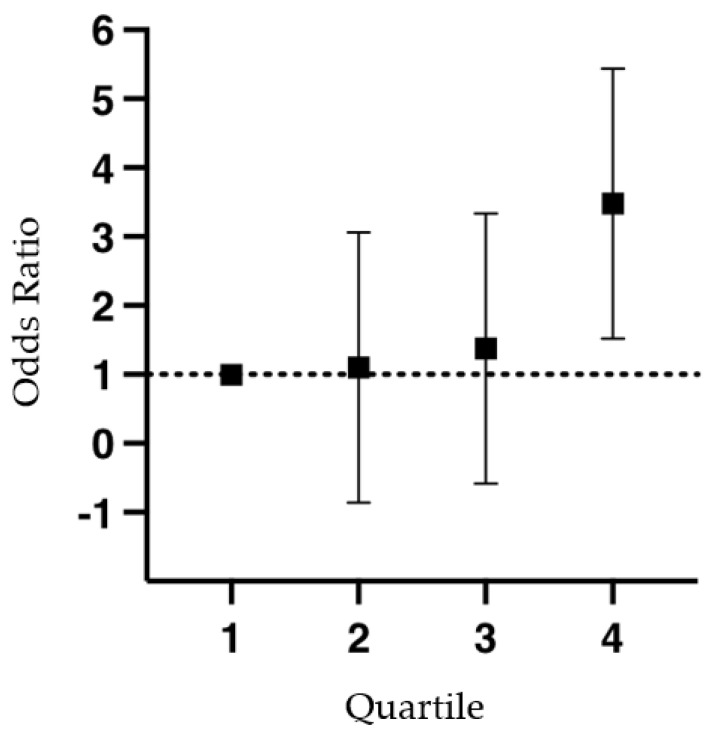
Odds ratio for thrombosis according to the Factor XI level.

In the cases, blood samples were collected after a mean (±SD) of 6 ± 1 months from the DVT diagnosis. D-dimer levels (mean ± SD) were significantly higher in cases (648 ± 143 ng/mL) than in controls (221 ± 106 ng/mL, *p* = 0.04). Moreover, both FVIII and fibrinogen levels (mean ± SD) were significantly higher in cases (267 ± 191% and 492 ± 113 mg/dL, respectively) than in controls (143 ± 54%, *p* = 0.01 and 305 ± 87 mg/dL, *p* = 0.01, respectively). The mean (±SD) FXI level among cases was 119.5 ± 25.6% (range 74.5–250.3%) vs. 106.3 ± 15.5% (range 78.0–148.1%) in controls (*p* < 0.001). Among the cases, no significant difference in FXI levels was found between males (116.8 ± 24.1%) and females (123.4 ± 27.3%, *p* = 0.1). Moreover, a significant linear correlation was observed between age and BMI vs. plasma FXI levels (*p* < 0.001 in both comparisons). The mean (±SD) plasma FXI level in patients with unprovoked DVT (117.5 ± 24.4%) was similar to that observed in patients with provoked DVT (119.1 ± 27.9%, *p* = 0.6). No significant difference in FXI levels was found between thrombophilic (114.4 ± 27.6%) vs. non thrombophilic patients (122.8 ± 25.3%, *p* = 0.3). The 90th percentile of the FXI levels in the control group was 130.1%. Among the 200 cases, 47 (24%) had values above this cutoff, compared with 20 (10%, by definition) in the control group (Table 2). The odds ratio for thrombosis, compared with patients in the first quartile (Q1), progressively increased with the FXI level: (a) Q1 vs. Q2 (FXI ≤ 96.5%) OR 1.1 (95% CI, 0.6 to 2.1, *p* = 0.48); Q1 vs. Q3 (FXI ≤ 104.8%) OR 1.4 (95% CI, 0.7 to 2.4, *p* = 0.61); and Q1 vs. Q4 (FXI ≤ 114.7%) OR 3.4 (95% CI, 1.9 to 6.0, *p* = 0.02) (Figure 2). The adjusted odds ratio for DVT in patients with an FXI level above the 90th percentile compared with those below was 2.4 (95% CI, 1.3 to 5.5, *p* < 0.001) (Table 2).

### 3.2. T2—At Least Three Months after T1

Among the 47 patients with plasma FXI levels above the 90th percentile, 4 were lost during follow-up. Thus, 43 patients (M/F 22/21, age range 31–87 years) were retested a mean (±SD) of 4 *±* 1 months after T1. None of the patients enrolled experienced recurrent VTE between T1 and T2. The main characteristics of the study population considered at T2 are reported in Table 3. No significant difference was found with regard to age, sex, and BMI between patients with plasma FXI levels >90th vs. ≤90th percentile (*p* > 0.05 in all comparisons). The mean (±SD) D-dimer, FVIII, and fibrinogen levels in patients with plasma FXI levels >90th were similar to those observed in patients with plasma FXI levels ≤90th percentile (*p* > 0.05 in all comparisons). The prevalence of thrombophilia was lower in patients with plasma FXI levels >90th vs. ≤90th percentile (31% vs. 51%) without reaching statistical significance (*p* = 0.42). The most common thrombophilia observed in the two subgroups was prothrombin G20210A mutation. Among the patients (n 16, 37%) who maintained high plasma FXI levels (>90th percentile of controls), the mean (±SD) value was 152.9 ± 28.9% (range 130.3–250.3%). The adjusted odds ratio for DVT in patients with persistently high FXI levels compared with those who had a lower level (≤90th percentile value) was 5.2 (95% CI, 2.3 to 13.2, *p* < 0.001) (Table 2).

## 4. Discussion

Our study showed a significantly higher risk of developing DVT in subjects with increased FXI levels (i.e., >90th percentile measured in a healthy control population). Interestingly, there was a positive linear correlation between FXI levels and thrombotic risk. We also observed a twofold increased DVT risk in the subgroup of patients with persistently—i.e., in two consecutive determinations made at least three months apart—increased FXI levels.

Overall, our findings are in line with previous studies in the literature. In fact, Meijers JCM et al. [6] reported an increased risk of developing venous thrombosis in subjects with increased plasma FXI levels, with an adjusted OR of 2.2 (95% CI, 1.5 to 3.2) similar to that observed in our study (OR 2.4, 95% CI 1.3 to 5.5). Moreover, in accordance with the findings of Meijers JCM et al. [7], the DVT risk associated with elevated FXI levels was shown to be dose-dependent. However, the relationship between increased FXI levels and risk of venous thrombosis was non-linear, being significantly greater in patients with highly elevated (above the 90th percentile) plasma FXI levels. Furthermore, we corroborated the previous findings in the literature on a possible association between high levels of FXI and increased thrombotic risk. In particular, we observed that 24% of patients with thrombosis in our cohort had elevated plasma FXI levels, a finding very similar to that observed by Meijers JCM et al. [7] who reported a 19% prevalence of subjects with increased FXI levels.

Although increased plasma FXI levels may be a key risk factor for developing thrombotic disease, it remains nonetheless very challenging to ascertain the causal nexus between the two events. Firstly, the molecular mechanisms through which elevated FXI levels promote thrombogenicity have not been defined, though we postulate that it may linked to the activation of prothrombin into thrombin. The sustained generation of thrombin may then contribute to the formation of a thrombus via two plausible mechanisms: (a) the conversion of fibrinogen to fibrin; (b) the inhibition of fibrinolysis by activating TAFI [25]. Secondly, plasma levels of coagulation factors vary wildly over time. In fact, coagulation factors often behave like acute phase proteins and their increase is unpredictable and transient. Furthermore, bearing in mind that most studies on this topic were conducted retrospectively, we cannot exclude the potential influence of a previous thrombotic event on plasma FXI levels. Thirdly, the lack of a consensus on cut-off values makes the use of increased FXI plasma levels as a thrombophilic marker problematic. Altogether, there are still too many unanswered questions to recommend the measurement of FXI plasma levels in routine thrombophilia screening panels.

Notably, our study revealed a novel finding that is, a further increase in thrombotic risk in subjects with persistently elevated levels of FXI over time. Given the high inter-individual variability that characterises plasma levels of coagulation factors, identifying a subgroup of patients with persistently high levels of FXI suggests that there may be underlying genetic factors. Nevertheless, the molecular basis that may contribute to the persistent increase in plasma FXI levels remains unknown. In particular, studies that attempted to identify the genetic determinants (e.g., polymorphisms in the *F11* gene) of increased FXI levels gave contradictory results. [26,27,28,29]. Regarding *F11* rs2289252, Manco L et al. [28] found no significant association (OR, 1.09; 95% CI, 0.75–1.59), whereas, using the same SNP, Bruzelius M et al. [29] found a significant association (HR, 1.8; 95% CI, 1.1–3.0).

In recent years, the central role of increased plasma FXI levels, and more generally the role of the activation of the intrinsic pathway, in the development of thrombotic disease, has led pharmaceutical companies to develop numerous molecules with anticoagulant activity that are capable of selectively inhibiting the function of FXI and FXIa [30]. The inhibition of FXI/FXIa may constitute a safe and effective anticoagulation strategy. Indeed, one of the main distinctive and peculiar features of all these drugs is that they appear to significantly reduce the thrombotic risk with an excellent safety profile in regard to possible bleeding complications [31]. The three approaches currently proposed are: monoclonal antibodies (mAbs), small molecules, and antisense oligonucleotides (ASOs) [32]. The strategy related to the use of mAbs (i.e., abelacimab, osocimab, BAY1831865, AB023/xisomab 3G3) is based on the interaction of specific humanised antibodies with different domains of FXI and/or FXIa, thus inhibiting the procoagulant function of these factors. Small molecules (i.e., asundexian and milvexian) are chemically synthesised compounds able to inhibit FXI and or FXIa with high affinity. Finally, ASOs (e.g., fesomersen) are able to target FXI mRNA in the liver thus preventing the synthesis of coagulation factors. To date, results have been published for phase 1 and 2 trials across a variety of clinical conditions, including VTE prophylaxis in patients undergoing knee arthroplasty, stroke prevention in AF, after MI, and after nonembolic stroke [33]. The results of a recently published meta-analysis showed that FXI inhibitors have a lower rate of bleeding compared with enoxaparin and with direct oral anticoagulants [34].

We would be remiss not to mention some of the limitations of our study. It was a monocentric case–control study with a relatively small sample size. Although this allowed us to enrol a homogeneous cohort of patients and to apply the same laboratory procedures to all patients for the determination of coagulation parameters, the small sample size barred us from including some risk factors (e.g., type of thrombosis—unprovoked or provoked) which could influence the levels of FXI in the multivariate analysis. This aspect is even more relevant considering that we were not able to carry out a detailed statistical analysis on patients (only n 16) who exhibited persistently high levels of FXI. Another limitation was the arbitrary decision to use the 90th percentile calculated in the control group to define subjects with high levels of FXI. The lack of consensus among experts on a cutoff makes it difficult to compare data from different studies. Finally, we performed follow-up imaging (T2) only in patients who presented with clear signs of VTE or post-thrombotic syndrome.

## 5. Conclusions

Our study concluded that increased FXI levels may be considered a dose-dependent risk factor for deep vein thrombosis. Interestingly enough, the persistence of high levels of FXI further increases said risk. Larger prospective studies are needed to confirm our results. It will also be interesting to ascertain the genetic markers associated with a persistent increase in FXI levels.

## Figures and Tables

**Table 1:** Characteristics of patients with venous thrombosis and controls.

	Patients (*n* = 200)	Controls (*n* = 200)
Age (yr)		
Mean	57.4	55.6
Range	26–87	25–89
Sex (M/F)	114/86	114/86
Body Mass Index (Kg/m^2^)		
Mean	27	24
Obese, BMI ≥ 30 (no.)	56	34
Thrombophilia		
FVIII, >250%	5	3
Fibrinogen, >600 mg/dL	9	5
Protein C, <60%, (no.)	8	4
Protein S, <60% (no.)	7	6
Antithrombin, <70% (no.)	4	2
Factor V Leiden (no.) ^†^	14	6
Prothrombin G20210A (no.) ^†^	11	7

^†^ All carriers were heterozygous.

**Table 2:** Risk of thrombosis according to FXI levels.

Factor XI *	Patients	Controls	Odds Ratio (95% CI)	Adjusted Odds Ratio ^‡^ (95% CI)
Study population				
≤90th percentile	153	180	1.0	1.0
>90th percentile	47	20	2.8 (1.6–4.9) ^#^	2.4 (1.3–5.5) ^#^
Patients with persistently increased FXI levels				
≤90th percentile	27	180	1.0	1.0
>90th percentile	16	20	5.3 (2.5–11.5) ^#^	5.2 (2.3–13.2) ^#^

* The 90th percentile of FXI levels in controls was 130.1%. **^‡^** Odds Ratio was adjusted for body mass index and other thrombophilias. ^#^ *p* value < 0.001. CI, confidence interval.

**Table 3:** Characteristics of patients at T2 (retested at least three months after T1).

	FXI > 90th Percentile (*n* = 16)	FXI ≤ 90th Percentile (*n* = 27)
Age (y)		
Mean	58.1	57.4
Range	31–87	26–85
Sex (M/F)	7/9	15/12
Body Mass Index (Kg/m^2^)	4	
Mean	26	23
Obese, BMI ≥ 30 (n)	26	3
Thrombophilia		
FVIII, >250%	1	1
Fibrinogen, >600 mg/dL	1	1
Protein C, <60%, (n)	0	2
Protein S, <60% (n)	0	1
Antithrombin, <70% (n)	0	0
Factor V Leiden (n)	1	4
Prothrombin G20210A (n)	2	5

## Data Availability

The data that support the findings of this study are available from the corresponding author upon reasonable request.
